# Optical Microbottle Resonators for Sensing

**DOI:** 10.3390/s16111841

**Published:** 2016-11-02

**Authors:** Pablo Bianucci

**Affiliations:** Department of Physics, Concordia University, Montral, QC H4B 1R6, Canada; pablo.bianucci@concordia.ca; Tel.: +1-514-848-2424 (ext. 3352)

**Keywords:** bottle resonators, whispering gallery modes, optical resonators, label-free sensing, optical sensing

## Abstract

Whispering gallery mode (WGM) optical microresonators have been shown to be the basis for sensors able to detect minute changes in their environment. This has made them a well-established platform for highly sensitive physical, chemical, and biological sensors. Microbottle resonators (MBR) are a type of WGM optical microresonator. They share characteristics with other, more established, resonator geometries such as cylinders and spheres, while presenting their unique spectral signature and other distinguishing features. In this review, we discuss recent advances in the theory and fabrication of different kinds of MBRs, including hollow ones, and their application to optofluidic sensing.

## 1. Introduction

**Figure 1:**
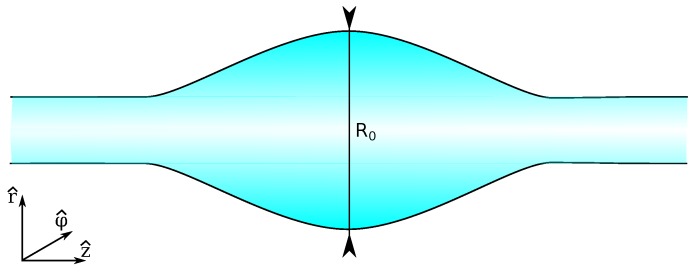
Schematic of a bottle resonator geometry.

Optical microresonators are microscopic structures capable of confining light in small spaces [1]. The optical confinement leads to the emergence of resonant electromagnetic modes, which show as narrow lines in their spectra. One of their most promising applications is that of label-free sensing, due to the sensitivity of the optical modes to external perturbations. In general, the sensitivity of resonant devices increases as the linewidth of their resonant features decreases. This linewidth is proportional to the optical losses, and it is inversely proportional to the *Q*-factor (a quantity defined as $Q=\lambda_{0}/\Delta \lambda$, where $\lambda_{0}$ is the center wavelength of the resonance, and Δλ is its linewidth). Whispering gallery mode (WGM) microresonators, where light is confined by total internal reflection in a dielectric structure with an axisymmetric cross-section, are the ones that have shown the lowest intrinsic losses [2]. Thanks to these low losses, they have the potential for extremely sensitive detection [3]. There are many different WGM geometries in use, such as spheres, cylinders, disks, rings, tubes, etc. The current record for *Q*-factor in optical microresonators is on the order of $10^{11}$ for crystalline CaF${}_{2}$ WGM resonators [4], while for amorphous silica (a very popular material due to its low cost and simplicity of processing) it is on the order of $10^{10}$ for microspheres in the visible range [5], $10^{9}$ for microspheres in the near infrared [6], and $10^{9}$ for planar structures like wedged microdisks [7]. Integrated on-chip microring resonators have shown record *Q*-factors of $10^{7}$ in silicon nitride waveguide platforms [8]. The most commonly used WGM microresonators for sensing are microspheres, due to their straightforward fabrication and very high *Q*-factors. Another less explored geometry is the microbottle resonator (MBR). A microbottle is a deformation of a cylinder (or tube) along its long axis, where the radius increases smoothly up to a maximum, to then decrease again (see Figure 1 for an illustration). Microbottles combine WGMs with “bouncing ball” modes, featuring caustics that limit the propagation about the axial direction, with routinely achievable *Q*-factors on the order of $10^{7}$.

The idea of using a thickening of the radius of a cylinder to fully confine light was brought up by Sumetsky [9], who called the geometry a microbottle, inspired by the geometric similarities to the magnetic “bottles” used to confine hot plasmas. Optical microbottle resonators have some advantages over microspheres, such as better control over the coupling using tapered optical fibers [10], fast tunability by the application of strain [11], and the possibility of obtaining a large number of equally-spaced modes in the spectrum [12].

Microbottles have not been used significantly as sensing devices where the medium to be analyzed lies outside the resonator. The recently introduced hollow MBRs are starting to shine in the context of optofluidic microresonator sensing [13]. An optofluidic microresonator is just a WGM microresonator that is hollow, so that a fluid can be run through it. The properties of the resonator change due to the fluid, and those changes can be detected by light that is coupled on the outside, away from the liquid. Optofluidic resonators simplify the delivery of fluids to the active resonator area, while also avoiding unwanted interactions between the probing light and fluid.

## 2. Theory of Bottle Resonators

Bottle resonators are WGM resonators, essentially cylindrically symmetric dielectric structures possessing a uniform dielectric constant (we are ignoring the effects of material dispersion). The radius changes (symmetrically with respect to the origin in an ideal bottle) with the axial position, as R(z). As long as the variation in radius is very small (i.e., dR/dz<<1), then the mode structure can be studied analytically. The first solution for the eigenfrequencies of bottle resonators was found by taking the Wentzel–Kramers–Brillouin (WKB) approximation [9], but we will use a method typically used for WGM resonators (which also gives explicit expressions for the fields) [10]. We can start from Maxwell’s equations in a uniform, source free, medium for harmonic fields oscillating with an angular frequency *ω*.

(1) $$\begin{array}{c} \begin{array}{cc} \vec{\nabla }\cdot \vec{D}=0, & \vec{\nabla }\times \vec{E}=i\omega \vec{B},\\ \vec{\nabla }\cdot \vec{B}=0, & \vec{\nabla }\times \vec{H}=-i\omega \vec{D}. \end{array} \end{array}$$

Taking the curl of the curl equations, using vector calculus identities and the constitutive relations $\vec{D}=\epsilon \vec{E}$ and $\vec{B}=\mu \vec{H}$, we can obtain two uncoupled Helmholtz equations for the electric ($\vec{E}$) and magnetic ($\vec{H}$) fields:

(2) $$\begin{array}{ccc} \nabla^{2}\vec{E}+k^{2}\vec{E} & = & 0, \end{array}$$

(3) $$\begin{array}{ccc} \nabla^{2}\vec{H}+k^{2}\vec{H} & = & 0, \end{array}$$

where $k^{2}=\mu \epsilon \omega^{2}$. While the equations are uncoupled, the fields are still coupled by the transversality requirement that holds in a uniform source-free geometry,

(4) $$\omega \mu \vec{H}=\vec{k}\times \vec{E},$$

where $\vec{k}$ is the wavevector with magnitude *k* pointing along the direction of propagation. It is possible, in some cases, to solve these equations including full polarization information, but it is simpler to take advantage of the bottles’ quasi-cylindrical geometry and focus on the experimentally-relevant modes whose electric field is oriented along the long axis of the bottle (so that $\vec{E}=E\;\hat{z}$, usually denoted as TE polarization). Under this condition, Equation (2) reduces to a scalar Helmholtz equation,

(5) $$\nabla^{2}E+k^{2}E=0,$$

and the magnetic field can be calculated (if desired) from the transversality Equation (4).

Equation (5) can be solved in cylindrically-symmetric systems by separation of variables, assuming that $E\left(r,\varphi ,z\right)=f\left(r,z\right)e^{im\varphi }$ (*m* is an integer azimuthal mode number). Thanks to the slow variation of the radius, it is a good approximation to separate on the radial and axial coordinates, f(r,z)=F(r)Z(z), so that,

(6) $$E\left(r,\varphi ,z\right)=F\left(r\right)Z\left(z\right)e^{im\varphi }.$$

Putting Equation (6) back into Equation (5) we find two separate equations for the radial and axial dependence,

(7) $$\begin{array}{c} \frac{d^{2}R}{dr}+\frac{1}{r}\frac{dR}{dr}+\left[k_{⊥}^{2}\left(z\right)-\frac{m^{2}}{r^{2}}\right]R=0, \end{array}$$

(8) $$\begin{array}{c} \frac{d^{2}Z}{dz^{2}}-k_{⊥}^{2}\left(z\right)Z=-k^{2}Z, \end{array}$$

where $k_{⊥}$ and $k_{z}$ are the transverse and axial components of the wavevector, satisfying that,

(9) $$k_{⊥}^{2}\left(z\right)+k_{z}^{2}\left(z\right)=k^{2}.$$

These equations are still coupled due to the *z*-dependency of the wavevector components, so we need to finish uncoupling them. This is simplest to do if we restrict ourselves to “fundamental” WGM modes, the ones with a trajectory lying closest to the surface. Under this condition, we can neglect the radial component of the wavevector; thus, $k_{⊥}=k_{\varphi }$, and

(10) $$k=\sqrt{k_{\varphi }^{2}+k_{z}^{2}}=\frac{2\pi n}{\lambda_{0}},$$

where $\lambda_{0}$ is the vacuum wavelength and *n* the resonator’s index of refraction.

Due to the symmetry of revolution, the z-component of the angular momentum should be conserved, requiring that the product $k_{\varphi }\left(z\right)R\left(z\right)$ is constant. Since we also know that at the caustics, at $z=\pm z_{c}$, there is no propagation along the *z* axis, then $k_{\varphi }\left(\pm z_{c}\right)=k$, and

(11) $$k_{\varphi }\left(z\right)=\frac{kR_{c}}{R(z)}=\frac{m}{R(z)},$$

where $R_{c}=R\left(\pm z_{c}\right)$ is the radius of the microbottle at the caustics, and $kR_{c}=m$. We can use these relations in Equation (8), obtaining now the equation for the axial dependence in terms of the microbottle profile R(z),

(12) $$\begin{array}{c} \frac{d^{2}Z}{dz^{2}}-{\left[\frac{m}{R(z)}\right]}^{2}Z=-k^{2}Z, \end{array}$$

Equation (12) is a Schrödinger-like equation,

(13) $$-\frac{d^{2}Z}{dz^{2}}+V_{\mathrm{eff}}\left(z\right)Z=E_{\mathrm{eff}}Z,$$

with

(14) $$V_{\mathrm{eff}}\left(z\right)={\left[\frac{m}{R(z)}\right]}^{2},\;E_{\mathrm{eff}}=k^{2}.$$

An analytic solution can be found to this equation if we choose a quasi-parabolic spatial profile,

(15) $$R\left(z\right)=\frac{R_{0}}{\sqrt{1+(\alpha^{2}z^{2})}}\approx R_{0}\left(1-\frac{\alpha^{2}z^{2}}{2}\right).$$

Then, Equation (13) becomes that of a simple harmonic oscillator with an energy shift $m^{2}/R_{0}^{2}$,

(16) $$-\frac{d^{2}Z}{dz^{2}}+\left(\frac{1}{2}\Delta E_{m}z^{2}+\frac{m^{2}}{R_{0}^{2}}\right)=k^{2}Z,$$

where $\Delta E_{m}=\sqrt{2}m\alpha /R_{0}$.

The possible wavevectors are now given by the eigenenergies of the harmonic oscillator plus the energy shift:

(17) $$k_{mq}^{2}=\frac{m^{2}}{R_{0}^{2}}+\left(q+\frac{1}{2}\Delta E_{m}\right),$$

where *q* represents a natural axial mode number. The corresponding solution for the *z*-dependence of the field is then given by:

(18) $$Z_{m}q\left(z\right)=C_{mq}H_{q}\left(\sqrt{\frac{\Delta E_{m}}{2}z}\right)e^{-\frac{\Delta E_{m}}{4}z^{2}},$$

with $H_{q}\left(x\right)$ is the Hermite polynomial of degree *q* with a normalization constant $C_{mq}={\left\{\Delta E_{m}/\left[\pi 2^{2q+1}{\left(q!\right)}^{2}\right]\right\}}^{1/4}$.

From the above discussion, it can be seen that the profile of the microbottle will directly influence the *z*-dependence of the electric field, and the spectral distribution of the different axial modes. A slowly-changing parabolic profile for the microbottle will then result in approximately equispaced axial resonant modes in its spectrum.

The solution is not yet complete, as we still need to solve for the the radial dependence. Thanks to Equation (10), we can rewrite Equation (7) as a Bessel differential equation with a parametric dependence on *z* via $k_{\varphi }\left(z\right),$

(19) $$\frac{d^{2}R}{dr}+\frac{1}{r}\frac{dR}{dr}+\left[k_{\varphi }^{2}-\frac{m^{2}}{r^{2}}\right]R=0.$$

The radial dependence will then be given by Bessel functions of the first, second, or third kind. The details will depend on the number of interfaces between the resonator and the outside medium; that is, whether the microbottle is solid or hollow.

### 2.1. Solid Microbottles

For a solid microbottle, there is a single interface, so (for a given azimuthal order *m*) the solution can be separated in two parts—one inside the bottle and one outside, assuming a refractive index $n_{o}$ for the medium:

(20) $$R\left(r,z\right)=\left\{\begin{array}{cc} A_{m}J_{m}\left(k_{\varphi }r\right), & r\leq R(z)\\ H_{m}^{\left(1\right)}\left(\frac{n_{o}}{n}k_{\varphi }r\right)+B_{m}H_{m}^{\left(2\right)}\left(\frac{n_{o}}{n}k_{\varphi }r\right), & r>R(z) \end{array}.\right.$$

On the inside, the field is proportional to a Bessel function (to avoid a singularity at the origin), while on the outside, it is a linear combination of Hankel functions of the first and second kind, which asymptotically approach outgoing and incoming cylindrical waves at very large distances, respectively. Requiring continuity of the field and its derivative for each R(z), we can obtain expressions for the coefficients.

### 2.2. Hollow Microbottles

In the case of a hollow (optofluidic) microbottle, there are two interfaces—an inner one at $R_{i}\left(z\right)$, and an outer one at R(z). That means that the solution is split in three pieces for the inside medium (with refractive index $n_{i}$), the resonator (*n*), and the outside medium ($n_{o}$), respectively:

(21) $$R\left(r,z\right)=\left\{\begin{array}{cc} A_{m}J_{m}\left(\frac{n_{i}}{n}k_{\varphi }r\right), & r\leq R_{i}\left(z\right),\\ B_{m}J_{m}\left(k_{\varphi }r\right)+C_{m}H_{m}^{\left(1\right)}\left(k_{\varphi }r\right), & R_{i}\left(z\right)<r\leq R\left(z\right)\\ H_{m}^{\left(1\right)}\left(\frac{n_{0}}{n}k_{\varphi }r\right)+D_{m}H_{m}^{\left(2\right)}\left(\frac{n_{0}}{n}k_{\varphi }r\right), & r>R(z), \end{array}.\right.$$

The inner field is, again, proportional to a Bessel function, and the outer field is again proportional to a linear combination of Hankel functions. The resonator field now has both Bessel and Hankel components, with coefficients to determine from requiring continuity of the field and its derivatives at both interfaces.

### 2.3. Surface Nanoscale Axial Photonics

A more recently introduced resonator (which can be considered as a microbottle with an extremely small variation of radius) is the surface axial photonics resonator (SNAPR). First proposed by Sumetsky [14], SNAPRs take advantage of the residual strain remaining after an optical fiber is pulled. When the fiber is locally annealed after being pulled, the release of this strain creates a sub-nanometer variation of the resonator effective radius, and creates axial confinement. While the theory being discussed here can be applied to SNAPRs, a full theoretical description that includes the coupling of light to and from waveguides can be found at [15].

## 3. Fabrication and Characterization

### 3.1. Solid Microbottles

The great majority of microbottle resonators are based on optical fibers; thus, their fabrication tends to be straightforward, without requiring microfabrication equipment. This simplicity is greatly appreciated in a research environment, but it implies that the fabrication throughput is low. In general, fiber-based microbottles have not yet been shown to be amenable to mass-production. The initial method used for their fabrication involves thermal softening and pulling. This involves heating up an optical fiber until its softening point (1665 ${}^{\circ }$C, generally done using a flame [16], a CO${}_{2}$ laser [17], or a ceramic microheater [18]) and then mechanically pulling it to decrease its diameter. First, the fiber is pulled to a desired starting diameter, and then a localized source of heat is applied with further pulling to create a constriction. This step is repeated at another location, resulting in the creation of a microbottle resonator that can be seen as a bulge in the optical fiber. Initial results showed low *Q*-factors [19], but *Q*-factors on the order of $10^{7}$ and $10^{8}$ became achievable with technical improvements [11,20].

Instead of heating and pulling, it has been shown that it is possible to obtain microbottles reversing the mechanical motion; that is, with a “soften and compress” method [21].

In this method, a continuous piece of fiber is heated in a controlled fashion while it is being compressed. The compression of the softened silica then results in a microbottle with good control of the geometry (defined by the compression and heating parameters). *Q*-factors close to $10^{6}$ have been obtained with this method. This technique has the advantage of being able to be implemented in a straightforward fashion in most commercially available fiber-optic fusion splicers, reducing the need for custom motion stages in a microbottle fabrication setup, but it can be problematic to implement if the desired diameter of the microbottle is significantly smaller than that of standard optical fibers.

Glass is not the only material that has been used to fabricate microbottles. It has been shown that ultraviolet (UV)-curing epoxy can be used to form droplets on an optical fiber that, thanks to interfacial forces, self-assemble into microbottles [22]. These resonators showed *Q*-factors near $10^{5}$, and a strong dependence of the resonance wavelengths on the power used to probe them (due to the large thermo-optic coefficient of the resin). A similar process, using a spin-on-glass instead of a resin resulted in the demonstration of an erbium-doped microbottle laser [23].

Finally, an alternative way to obtain bottle-like confinement is by creating a localized change in the refractive index of a dielectric cylinder, generally induced by the photo-refractive effect. This has been demonstrated on chalcogenide fibers [24], since chalcogenide materials possess a strong photosensitivity.

**Figure 2:**
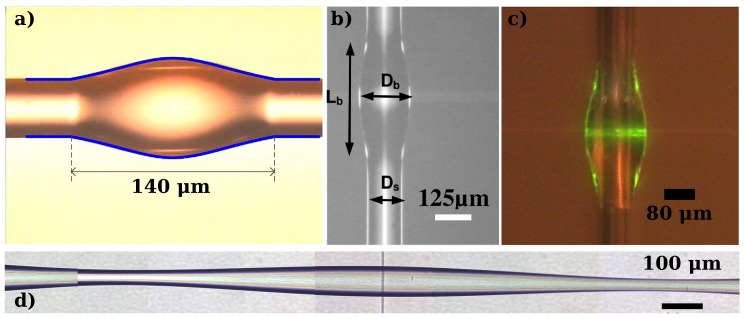
Microscope images of microbottles fabricated with different methods. (**a**) Epoxy-based microbottle. Image adapted with permission from [22] (©the Optical Society of America); (**b**) “soften-and-compress” method. Image adapted with permission from [21] (©the Optical Society of America); (**c**) spin-on-glass-based microbottle, image adapted from [23], released under the CC BY 4.0 license (http://creativecommons.org/licenses/by/4.0/); (**d**) “heat-and-pull” method.

Figure 2 shows different solid microbottles fabricated using the described methods.

The fabrication of the above-mentioned SNAPRs consists of a localized treatment that creates a sub-nm change in a fiber’s effective radius. The localized treatment can be either thermal (with a CO${}_{2}$ laser [25] or a flame) or photo-induced, exposing a photo-sensitive fiber to UV radiation with an appropriate mask [26].

### 3.2. Hollow Microbottles

**Figure 3:**
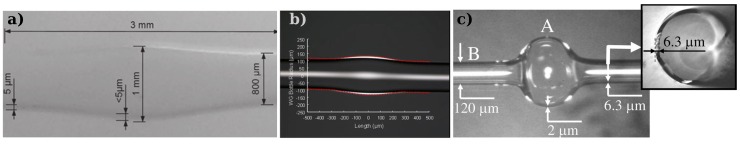
Optical microscope images of hollow microbottles fabricated using different methods; (**a**) heating a pressurized thin capillary. Image reprinted with permission from [31] (©IOP Publishing. All rights reserved); (**b**) “soften-and-compress” method used with a capillary. Image reprinted with permission from [29] (©The Optical Society of America); (**c**) reduction of a capillary’s diameter on the sides using a CO${}_{2}$ laser. Image reprinted with permission from [14] (©The Optical Society of America).

The fabrication of hollow microbottles is similar to that of solid ones, except that the starting stock is not a solid cylinder but rather a capillary, and care must be taken not to collapse the hollow interior (this is generally done by sealing one end of the the capillary and pressurizing it during the fabrication). Hollow microbubbles (large bottles that can almost be considered as spheres) were first demonstrated using a CO${}_{2}$ laser to reduce the diameter of a capillary tube on both sides of the bubble [14]. This technique was used to create an optomechanical microbottle capable of mechanical interactions with a fluid circulating within it [27]. The uniformity of the resulting microbottles is improved when using two CO${}_{2}$ lasers [28]. The “soften-and-compress” [21] method has also been applied to the fabrication of hollow microbottles, adding a moderate pressurization of the capillary to the splicer-based method [29]. For sensing purposes, it might be desirable to make microbottles with very thin walls. This can be achieved by thinning the initial capillary by thermal pulling [30], or by etching the inside wall with hydrofluoric acid [13]. An even simpler fabrication process has been demonstrated, where a previously-thinned capillary is heated with a flame while it is pressurized with air [31]. As the silica softens, the air pressure expands it, forming a smooth bubble. Some of the microbottles fabricated with these methods can be seen in Figure 3. Hollow SNAPRs can be also fabricated by locally annealing a capillary with a CO${}_{2}$ laser or a flame.

## 4. Applications to Sensing

Whispering-gallery-mode resonators, with their high and ultra-high *Q*-factors, have been proposed as very sensitive refractometric, chemical, and biological sensors [3]. Microbottles, with ultra-high *Q*-factors, improved coupling to tapered fibers, and clean spectra are perfect devices for this application. Furthermore, hollow optofluidic microbottles are ideal for sensing either liquids or gases that flow through their inner channel. Their geometry allows for easy fluidic coupling (unlike other resonators, such as microspheres or microtoroids), as well as keeping the interrogating tapered fiber away from the fluid (thus preventing contamination and a decrease of the measurement signal) [32].

The most common method for detecting changes is by measuring resonance shifts. This change can come from simple changes in the refractive index of the surrounding medium, which makes microbottles excellent refractometers (with initial sensitivity of 0.5 nm/RIU [33], and more recently 18.8 nm/RIU [34] with a detection limit of $5.4\times 10^{-5}\;\mathrm{RIU}$), or from the presence of molecules or nanoparticles in the proximity of the resonator boundaries [35]. Resonance shifts are subject to noise (generally due to temperature or laser fluctuations), which reduces the detection limits [36]. It is possible to mitigate this noise by careful control of the resonator environment [37]. Even better is applying a self-referencing technique using two resonant modes, to reject the common noise [38]. Recent results using self-referencing have shown a noise-equivalent detection limit of 10 fg/mL [39] for bovine serum albumin (BSA, a standard for protein concentration) in water with a hollow microbottle. It is also possible to detect changes by monitoring the absorption (using the fractional depth change of a resonance), a method that is more resistant to fluctuations and that has shown the detection of methane in air at partial pressures as low as 0.1% [31].

The specific geometry of the resonator—particularly its wall thickness—can have an impact on the potential sensitivity limits. Based on finite-element-model simulations, it has been found that sensitivity is maximized for the “quasi-droplet" configuration, where the resonator shell is thin enough that the field extends significantly into the hollow core [40].

Another physical quantity that can be transduced to a resonant shift in hollow microbottles is the internal aerostatic pressure, since the resonant wavelength of the microbottle modes depend very sensitively on the microbottle geometry [41]. Using telecom wavelengths, demonstrations have shown a pressure sensitivity of 0.36 nm/bar in polymethyl meta-acrylate (PMMA) capillaries with thick (80 μm) walls [42], and a sensitivity of 0.15 nm/bar with thin-walled (500 nm) microbubbles [30]. The sensitivity can be improved by using shorter-wavelength light (780 nm), with a maximum resolution of 0.17 mbar [30]. Similarly, changes in temperature will also give rise to a shift in the resonances, which can be used as a thermometer, with a demonstrated sensitivity of up to 0.20 nm/K in an ethanol-filled microbubble [43].

A different transduction possibility is the use of optomechanical coupling to detect mechanical interactions between the liquid and the microbottle walls, thus making a quantifiable viscosity sensor [27,44,45]. This idea has been recently expanded to the detection of flowing particles (as in flow cytometry, with additional information available about their physical properties such as mass density, compressibility, and viscoelasticity), with potential rates as high as 10,000 particles per second [46].

Thanks to ultra-high *Q*-factors, it is also possible to detect the presence of nanoparticles inside the hollow WGM resonators. This can be done by either monitoring the resonant shifts (as theoretically analyzed in [47] for a variety of hollow geometries), or by measuring the mode-splitting caused by the nanoparticle-induced scattering of light [48]. This has been predicted to be able to detect polystyrene nanoparticles with radii as small as 3.1 nm in air [49].

Other schemes introduced that could be useful for sensing in hollow microbottles are Raman lasing [28], where the laser line narrowing increases the potential sensitivity, and nonlinear optical effects. Four wave mixing [50] and hyperparametric oscillations [51] have been reported in hollow microbubbles, generating comb-like spectra that could potentially be used for exciting new applications such as spectroscopic detection.

Finally, hollow resonators can go beyond simple detection, as there has been a proposal to use hollow SNAPRs to not only detect, but also manipulate particles flowing through the resonator thanks to optical forces [52].

## 5. Conclusions

From the previous discussion, it is clear that microbottle resonators possess a unique set of characteristics: simplicity of fabrication, simplified coupling, ability to obtain equispaced resonant modes, and tunability. Hollow microbottle resonators—as they are naturally optofluidic resonators—have the greatest potential as WGM-based sensing devices, since they allow for isolation of the coupling light and the liquid or gas to be measured. The demonstrations of their sensing capabilities have not reached their limit yet, as they have only very recently been introduced, but it is only a matter of time until they become a significant technology for optofluidic sensing.
